# Enhancing the Nutritional Profile of *Tenebrio molitor* Using the Leaves of *Moringa oleifera*

**DOI:** 10.3390/foods12132612

**Published:** 2023-07-06

**Authors:** Konstantina Kotsou, Theodoros Chatzimitakos, Vassilis Athanasiadis, Eleni Bozinou, Christos I. Rumbos, Christos G. Athanassiou, Stavros I. Lalas

**Affiliations:** 1 Department of Food Science and Nutrition, University of Thessaly, Terma N. Temponera Str., 43100 Karditsa, Greece; kkotsou@agr.uth.gr (K.K.); tchatzimitakos@uth.gr (T.C.); vaathanasiadis@uth.gr (V.A.); empozinou@uth.gr (E.B.); 2 Laboratory of Entomology and Agricultural Zoology, Department of Agriculture, Crop Production and Rural Environment, School of Agricultural Sciences, University of Thessaly, Phytokou Str., 38446 Volos, Greece; crumbos@uth.gr (C.I.R.); athanassiou@uth.gr (C.G.A.)

**Keywords:** edible insects, *Tenebrio molitor*, *Moringa oleifera*, proximate composition, carotenoids, vitamin A, vitamin C, proteins, fat, fatty acids

## Abstract

Nowadays, more and more research is being carried out on various feeds of *Tenebrio molitor* larvae, in order to increase their nutritional value and render them a valuable component of the human diet. In this study, *Moringa oleifera* leaves were used in different proportions (up to 50%) to substitute wheat bran (the usually employed feed), in order to evaluate their effect on the growth and development of the larvae, as well as on their composition in crude protein, fat and fatty acids, ash, vitamins, and antioxidants. It was found that the addition of *M. oleifera* leaves in the feed had no negative impact on the development and survival of the insects, while an increase in their nutritional value was recorded. More specifically, an increase in the crude protein of up to 22.61% and vitamin C and A contents of up to 40.74% and 491.63%, respectively, was recorded. Therefore, the use of *M. oleifera* leaves as a feed additive is highly recommended for rearing *T. molitor* larvae to enhance the nutritional value of the insects.

## 1. Introduction

*Tenebrio molitor* L. (Coleoptera: *Tenebrionidae*) (TM), commonly known as the yellow mealworm, has attracted much scientific and commercial attention in recent years, due to its nutritional value [1]. Due to their high content in protein, essential amino acids, fat, and essential fatty acids, TM larvae are considered to be a highly nutritious food [2,3,4]. The crude protein and fat content of the larvae vary depending on the rearing substrate and conditions. Generally, protein levels of 20 to 50% and fat levels of 20 to 45% have been reported [5]. The larvae are also rich in unsaturated fatty acids, including oleic acid, linoleic acid, and linolenic acid, with respective quantities of ~41, ~30, and ~1% [6]. In addition, TM larvae contain several essential amino acids, including isoleucine (~4%), tyrosine (~4%), phenylalanine (~3%), leucine (~3%), lysine (~3%), and methionine (~2%) [6]. However, despite their high nutritional content and ease of farming, TM larvae lack or contain low amounts of some essential nutrients, including vitamins A and C, and other bioactive compounds, such as polyphenols [7]. As such, the nutritional quality of TM larvae can be enhanced by increasing the content of essential nutrients, as well as polyunsaturated fatty acids and antioxidants.

*Moringa* species belonging to the *Moringaceae* family are widely imported to countries with mild climates [8]. Among the different species, *Moringa oleifera* (MO) is the most well-known [9]. MO is a highly nutritious multi-purpose plant with high nutritional values, each part of which can be used for either nutritional or commercial purposes [10,11,12]. Especially, the leaves of MO exhibit a range of health benefits upon consumption [13]. They are particularly rich in proteins, providing all of the essential amino acids required by the human body [14]. Also, they contain high levels of total polyphenols [15], flavonoids [16], and vitamins such as vitamins C and E [14], β-carotene [17], and ash [14]. The protein content of MO leaves reaches up to 26.3%, while polyphenols reach 4512.2 mg of gallic acid equivalents (GAE) per 100 g of dry matter (DM). The leaves also contain flavonoids such as myricetin, quercetin, and kaempferol. Vitamins C and E are present in concentrations of 203.1 and 104 mg/100 g DM, respectively, while β-carotene is present in a concentration of 2.02 mg/100 g DM [14]. Overall, the high nutritional value and beneficial properties of MO leaves make them a valuable addition to the human diet. Recent studies have also shown that MO has potential as a feed additive for livestock and poultry due to its high protein and mineral content [18,19]. However, there is limited research on the use of MO as a feed additive for insects, specifically for TM larvae.

The purpose of our study was driven by the growing interest in identifying sustainable and nutritious food sources to address global challenges such as food insecurity, environmental sustainability, and the increasing demand for protein-rich diets. TM larvae have gained attention as a potential solution due to their high nutritional value and low environmental footprint. However, in order to fully harness the benefits of mealworms as a viable food source, it is crucial to optimize their nutritional composition. This optimization can be achieved through appropriate feed formulation. Wheat bran is a commonly used feed for mealworms, but there is a need to explore alternative feed sources that can enhance their nutritional value. The aim of this study was to explore methods to increase the nutritional value of TM larvae, with the ultimate goal of enhancing their potential as a valuable component of the human diet. As such, we focused on the enhancement of the nutritional profile of TM larvae, with a particular focus on increasing the levels of crude protein, vitamins A and C, while at the same time achieving increased content of fats, fatty acids, ash, and antioxidants. This enrichment is achieved through natural means, by incorporating varying proportions of the MO leaves into the larval diet. In addition to enriching the nutritional composition, the study also evaluates the impact of MO leaves on the growth and survival of TM larvae. By accomplishing this dual objective, we not only strive to create nutrient-rich larvae but also ensure optimal larval growth.

## 2. Materials and Methods

### 2.1. Chemicals and Reagents

All solvents (hexane, acetone, ethanol, methanol) used were of HPLC grade and supplied by Carlo Erba (Val de Reuil, France). Gallic acid, sodium anhydrous carbonate, 2,2-diphenyl-1-picryl-hydrazyl (DPPH), 2,4,6-tri-2-pyridinyl-1,3,5-triazine (TPTZ) and Folin-Ciocalteu reagent were purchased from Penta (Prague, Czech Republic). Iron (III) chloride, hydrochloric acid, ascorbic acid, β-carotene, trichloroacetic acid, hydrochloric acid, and Bradford reagent were obtained from Sigma-Aldrich (Steinheim, Germany).

### 2.2. Insects and Plant Material

All experiments were conducted using TM individuals reared in the Laboratory of Entomology and Agricultural Zoology of the University of Thessaly. In order to obtain eggs and newly hatched larvae (7-days-old larvae), about 500 adults of TM were placed in trays (24 × 29.5 × 10 cm) with an opening on their top cover to allow air circulation. Trays were filled with 2000 g of white flour as oviposition substrate, whereas adults were placed on top of a mesh to prevent egg cannibalization. Agar was provided to adults *ad libitum* as a source of moisture. Insects were maintained under constant conditions, i.e., 26 ± 1 °C, 55 ± 5% relative humidity (r.h.), and continuous darkness.

MO leaves were collected from 2 months old plants cultivated in the Krya Vrisi area of Karditsa county (at 39°19′6.97″ N and 21° 52′39.16″ E) according to Google Earth. After washing with tap water and drying with paper towels, they were placed in a freeze-dryer (Biobase BK-FD10P freeze-dryer, Jinan, China) for 24 h and then crushed into powder and kept frozen (−20 °C) until further use.

### 2.3. Feeding Trials

In a series of feeding trials, we evaluated the growth of TM larvae on wheat bran-based feeding substrates with different percentages of dried MO leaves, i.e., 10% (MO10), 25% (MO25), and 50% (MO50). Wheat bran alone (MO0) served as control. Bran was procured from a local store in Volos, Greece, and had a particle size that did not exceed 2 mm.

In a first trial, 50 TM larvae were introduced in plastic cylindrical vials (6.5 cm diameter, 8.8 cm height; Carl Roth GmbH & Co. Kg, Karlsruhe, Germany) with a screened opening in their top cover to allow air circulation, together with 8 g of each substrate, having different vials for each substrate. Agar was provided to larvae as a moisture source three times per week. Additional feed was provided to the larvae when feed was almost depleted. To determine the larval survival and growth, larvae were separated from the substrate and counted, whereas their total weight was determined on a precision scale (Equinox EAB125i, Adam Equipment Inc., FoxHollow Road, Oxford, UK). The individual larval weight was calculated by dividing the total weight by the number of larvae. To determine the survival rate, the number of larvae at each evaluation point was divided by the initial number of larvae. The same procedure was repeated every week until the emergence of the first pupa in each vial. There were six vial replicates for each dietary treatment. During the bioassay, all insects were kept under the conditions described above.

In a second feeding trial, TM larvae were reared at a larger scale to produce enough material for the subsequent larval composition analyses. The same feeding substrates as described above were used in this trial, i.e., MO0, MO10, MO25, and MO50. Briefly, 2500 larvae were introduced into plastic trays (24 × 29.5 × 10 cm) together with 500 g of each substrate and reared for six weeks. Agar was provided to larvae *ad libitum* as a moisture source. After the 6-week interval, larvae were separated by sieving from the rearing substrate, fasted for 24 h, weighed, and euthanized by freezing (−20 °C). Afterward, larvae were placed in a freeze-dryer for 24 h and crushed into a fine powder that was stored in glass vials at −30 °C until further analysis.

### 2.4. Larval Composition Analysis

#### 2.4.1. Water Content Calculation

The determination of the moisture content of the larvae was carried out by a gravimetric method. The weight of the samples was recorded prior to and after the freeze-drying.

#### 2.4.2. Crude Protein Content

To extract proteins from each sample, 10 mL of distilled water at pH 12, in order to be secured alkaline environment (adjusted with NaOH—1M), was mixed with 1 g of the sample [20]. The extraction took place at room temperature for 60 min at 500 rpm and upon completion, the mixture was centrifuged for 5 min at 4500 rpm and the supernatant was retracted and transferred to another vial. To ensure total protein extraction, the extraction-isolation step was repeated two more times and the supernatants were mixed. The Bradford method was used to determine the crude protein content of the pooled sample. 900 μL of Bradford reagent were mixed with 100 μL of the sample and allowed to react for 10 min in the absence of light. In the end, the absorbance of the samples was measured at 595 nm using a spectrophotometer (Shimadzu UV-1700 Pharma Spectrophotometer, Kyoto, Japan). To quantify the amount of proteins, a standard calibration curve was prepared using bovine serum albumin.

#### 2.4.3. Carbohydrates

In order to extract and determine the amount of carbohydrates, the solvent:solute was mixed in a ratio of 1 g of dried larvae:10 mL of distilled water and dipped in an heated oil bath (50 °C) for 1 h. After the extraction was terminated, the mixture was centrifuged for 5 min at 4500 rpm and the supernatant was retracted. The carbohydrate content of the samples was determined according to a previously published method [21]. In a vial, 0.22 mL of the supernatant were transferred to a 1.5 mL Eppendorf tube and immediately concentrated in 0.65 mL of concentrated sulfuric acid with 0.13 mL of phenol solution (0.13 mL) (5% *w*/*v* in distilled water). The reaction took place in a water bath at 90 °C for 5 min and was then allowed to cool down in a water bath at cool temperature (20 °C) for another 5 min. The absorbance of the solution was measured at 495 nm using a spectrophotometer and a calibration curve was prepared using D(+)-glucose as a standard.

#### 2.4.4. Ash

The gravimetric approach was used for the determination of crude ash [20]. In a porcelain crucible, ~5 g of dry *T. molitor* powder was weighed, recorded and placed in an oven for 5 h. The temperature in the oven was increasing by 5 °C/min to 550 °C and the samples was heated until no black residue was observed. After cooling to room temperature, the sample was weighted and the ash content was calculated.

#### 2.4.5. Total Fat, Fatty Acids, and Calculated Oxidizability Value (COX)

The larval fat content was determined in percent (%) by the defatting procedure. In a 10 mL glass Duran, exactly 1 g of sample was weighed and 10 mL of hexane were added, as a degreasing agent. The mixture was stirred for 60 min at 40 °C at 600 rpm. After centrifugation for 2 min at 4000 rpm, the supernatant was poured into a pre-weighed flask and hexane removal was performed using a rotary evaporator. The defatting procedure was repeated three times on the solid residue. The procedure for the detection of fatty acids and their abundance in *T. molitor* larval samples was followed according to Athanasiadis et al. [22]. More specifically, fatty acid methyl esters were prepared by reaction of the oil (200 μL) with a small amount of sodium methoxide in methanol at room temperature. The reaction lasted about 5 min with gentle agitation of the mixture periodically. After the reaction was completed, 10 mL of diethyl ether was added, and the organic layer was washed three times with water, dried over Na_2_SO_4_, and reduced to 1 mL by heating in a water bath (40 °C). The solution obtained was then subjected to GC analysis. An Agilent Technologies (Santa Clara, CA, USA) Gas Chromatograph model 7890A, equipped with an Omegawax capillary column (30 m × 320 μm × 0.25 μm) (Supelco, Bellefonte, PA, USA), was used. Helium was the carrier gas at a flow rate of 1.4 mL/min. The column temperature program was: initially isotherm for 5 min at 70 °C, ramped to 160 °C at a rate of 20 °C/min, then increased to 200 °C at a rate of 4 °C/min and increased up to 240 °C at a rate of 5 °C/min. The injector and flame ionization detector (FID) temperatures were maintained at 240 and 250 °C, respectively. The flow rate for hydrogen was 50 mL/min, for air 450 mL/min, and the makeup flow of helium 50 mL/min. Samples of 1.0 μL were injected in split mode (1:100). The individual peaks were identified by comparison with reference standards from FAME Mix C8–C24 (Sigma-Aldrich, St. Louis, MO, USA).

In addition to the fatty acids and the PUFA:SFA, MUFA:PUFA and ω-6:ω-3 ratios, and calculated oxidizability (COX) value, four additional types known as index of atherogenicity (IA), index of thrombogenicity (IT), index of hypocholesterolemic/hypercholesterolemic (HH), and health-promoting index (HPI) were calculated. In more detail, the IA is the fatty acid atherogenesis and characterizes its potential [23]. IT characterizes the thrombogenic potential of fatty acids, indicating the tendency to form clots in blood vessels, and provides the contribution of different fatty acids, indicating the relationship between prothrombogenic fatty acids (C12:0, C14:0, and C16:0); and anti-thrombogenic fatty acids (MUFAs and the ω-3 and ω-6 families) [24]. The HH index is the ratio of hypocholesterolemic/hypercholesterolemic and it is described as a health promotion index to assess the nutritional value of dietary fat [25]. These indexes were calculated according to the following equations:

(1) $$\mathrm{COX}=\frac{1\;\left(18\text{:}1,\;\%\right)+10.3\;\left(18\text{:}2,\;\%\right)+21.6\;(18\text{:}3,\;\%)}{100}$$

(2) $$\mathrm{IA}=\frac{\left[C12\text{:}0+\left(4\;\times \;C14\text{:}0\right)+C16\text{:}0\right]}{\sum \mathrm{UFA}}$$

(3) $$\mathrm{IT}=\frac{\left(C14\text{:}0+C16\text{:}0+C18\text{:}0\right)}{[(0.5\;\times \sum \mathrm{MUFA})+(0.5\;\times \sum \omega -6)+(3\;\times \sum \omega -3)+(\omega -3/\omega -6)]}$$

(4) $$\mathrm{HH}=\frac{(C18\text{:}1+\sum \mathrm{PUFA})}{\left(C12\text{:}0+C14\text{:}0+C16\text{:}0\right)}$$

(5) $$\mathrm{HPI}=\frac{\sum \mathrm{UFA}}{\left[C12\text{:}0+\left(4\;\times \;C14\text{:}0\right)+C16\text{:}0\right]}$$

#### 2.4.6. β-Carotene—Vitamin A

To calculate the β-carotene and vitamin A content of the samples, the method described by Ocampo et al. [26] was employed. Extraction was carried out in an ice bath for 5 min with a solvent:sample ratio of 1:10 g respectively, using ethanol as solvent. The mixture was then centrifuged for 5 min at 4500 rpm and the absorbance of the extract was read at 450 nm.

A modified colorimetric analysis was used for the ascorbic acid content [20]. An amount of 1.0 g of powder from each *T. molitor* sample was mixed with 5.4 mL of distilled water: methanol mixture (60:40, *v*/*v*) and 0.6 mL of 10% *w*/*v* trichloroacetic acid solution. The solution was vigorously vortexed for 1 min, 4 mL of hexane was added and the resulting mixture was for 30 min. Next, the sample was centrifugated for 10 min at 10,000 rpm, and the lower aqueous phase was retracted. From this solution, 1 mL of the aqueous layer was diluted with 0.5 mL of Folin-Ciocalteu reagent (10% *v*/*v*) and allowed to incubate for 10 min. Finally, the absorbance was measured at 760 nm and quantification was performed by an ascorbic acid calibration curve.

#### 2.4.7. Determination of Total Polyphenol Content (TPC)

The extracted solution described in Section 2.4.6 was also used for the quantification of total polyphenol content (TPC). A method previously mentioned [27] was used, whereby 100 µL of the sample was mixed with 100 µL of Folin-Ciocalteu reagent and allowed to react for 2 min. Then, 800 μL of Na_2_CO_3_ solution (5% *w*/*v*) was added and the solution was incubated at 40 °C for 20 min. Finally, the absorbance was measured at 740 nm and a standard gallic acid calibration curve was used in order to find the exact amount of TPC. Essentially, the TPC of each sample was estimated as mg of gallic acid equivalents (GAE) per g dw, using the following Equation (6):

(6) $$Y_{\mathrm{TP}}\;(\mathrm{mg}\;\mathrm{GAE}/g\;\mathrm{dw})=\frac{C_{\mathrm{TP}}\;\times \;V}{w}$$

where *V* is the volume of extraction medium (in L) and *w* is the dry weight of the sample (in g).

#### 2.4.8. Iron Reduction Antioxidant Power Test (FRAP)

The sample prepared according to Section 2.4.6 was also used to determine the antioxidant capacity of *T. molitor* following a previous study [28]. Briefly, 0.10 mL of a mixture containing an equal amount of FeCl_3_ (4 mM in 0.05 M HCl) and the sample were incubated for 30 min at 37 °C. After incubation, 0.90 mL of TPTZ solution (1 mM in 0.05 M HCl) was added and allowed to react for 5 min. The absorbance of the solution was measured at 620 nm and quantified using a calibration curve of ascorbic acid as the standard compound. The results (*P*_R_) were expressed as μmol ascorbic acid equivalents (AAE) per g dw, using the following Equation (7):

(7) $$P_{R}\;(\mu \mathrm{mol}\;\mathrm{AAE}/g\;\mathrm{dw})=\frac{C_{\mathrm{AA}}\;\times \;V}{w}$$

where *V* is the volume of extraction medium (in L) and *w* is the dry weight of the sample (in g).

### 2.5. Statistical Analysis

A total of 8 measurements (four replicate trays and two subsamples per tray) were made for the larval survival and growth determination. All measurements for the proximate composition analysis were made in triplicates with three samples in each, yielding a total of nine measurements. Standard deviation (SD) was used to express results as the mean values of the nine measurements. The Kolmogorov–Smirnov test was then performed to see whether the data were normally distributed. The Kruskal–Wallis test was used to determine whether there were any statistically significant differences (*p* < 0.05) between the samples. SPSS (version 29) (SPSS Inc., Chicago, IL, USA) software was used to perform the statistical analyses.

## 3. Results and Discussion

In this study TM larvae were examined, so as to enhance their nutritional value. The selection of TM larvae was based on several reasons. Firstly, mealworms are relatively easy to rear, have a short life cycle, and can adapt to different environmental conditions [1]. These characteristics make them an ideal model insect species for studying nutritional improvements and optimizing rearing practices [3]. Secondly, TM larvae offer several practical advantages for research purposes. They have a relatively short life cycle, allowing for efficient and rapid experimentation. Their small size and ease of handling facilitate experimentation. Furthermore, TM larvae have been extensively studied and characterized, both in terms of their nutritional composition and rearing practices [3,4,5,6]. This existing knowledge base provides a solid foundation for conducting research on optimizing their nutritional value and investigating the effects of feed additives, such as MO leaves in our study. By selecting TM larvae, we aim to build upon the existing body of research and contribute to the scientific understanding of their potential as a sustainable food source.

### 3.1. Survival and Growth of TM Larvae

The survival rates of TM larvae over time fed on the evaluated substrates are presented in Table 1. High survival rates were recorded for the wheat bran control (94% at the end of the rearing). When bran was fortified with MO leaves larval survival was slightly reduced, however, differences were significant only at the final evaluation intervals (Weeks 5 and 6) and when MO leaves were added to the diet at high percentages, i.e., 25 and 50%. Nevertheless, larval survival in the different treatments was considerably high and was never lower than 84%. Previous studies have demonstrated the significant effect of diet on the survival of TM larvae [29,30,31]. For instance, significant differences were recorded in the survival of TM larvae fed on diets based on agricultural byproducts [30,31]. Indicatively, high larval mortality rates were recorded for TM larvae fed lucerne-including diets, which may be related to the presence of antinutritional factors present in this plant [31]. To our knowledge, no previous work investigated the effect of the addition of MO leaves in TM diets. When nymphs of the house cricket, *Acheta domesticus* (L.), and the field cricket, *Gryllus bimaculatus* De Geer (Orthoptera: Gryllidae), were reared on diets supplemented with 10% of MO leaves, mortality rates were significantly higher compared to the control diet for both insect species tested [32]. A recent study that evaluated the susceptibility of MO leaves to Pyralid moths showed that both the powdered and the dried MO leaves were not susceptible to moth attacks, as they could not support larval growth for most of the moth species tested and this finding was mainly attributed to the presence of secondary metabolites in the MO leaves [12,13,14,33].

**Figure 1:**
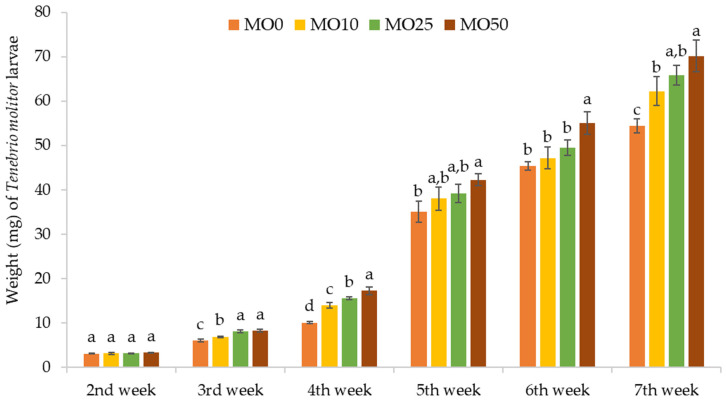
Individual larval weight (mg) of *Tenebrio molitor* larvae (±SD) fed for six weeks with wheat bran (control) (MO0) and bran fortified with different rates of MO leaves (10 (MO10), 25 (MO25), and 50% (MO50)) (*n* = 8). In all cases, values represent means ± SD (*n* = 8; df = 3; *p* = 0.05). Within each evaluation interval (Week 1, 2, 3, 4, 5, and 6), different letters denote statistically significant differences for *p* < 0.05.

The individual larval weight during the trial is shown in Figure 1. In general, larval growth was positively affected by the inclusion of MO leaves. More specifically, at the last evaluation point (Week 6) the weights of TM larvae fed on the diets fortified with different percentages of MO leaves were significantly higher than control (54 mg) and ranged between 62 (MO10) and 70 mg (MO50). Similarly, the inclusion of orange albedo into the diet of TM larvae exerted a positive effect on larval development, as it has been shown by a previous work [20]. Data on the effect of the addition of MO leaves into the diets of edible insects are limited. In one of the few studies available, the final weight of both *A. domesticus* and *G. bimaculatus* reared on feed supplemented with 10% MO leaves was significantly lower compared with the weight of larvae fed on the control feed, indicating a lower palatability for the MO feeds [33]. Overall, although the addition of the MO leaves powder into the diet of TM larvae marginally reduced larval survival, especially at high inclusion rates, it resulted in higher final larva weights, suggesting an improvement of the nutrient profile of the MO feeds compared to bran, which subsequently resulted in the increased larval growth and development.

### 3.2. Evaluation of the Nutritional Value of the Larvae

Prior to evaluating the larvae for their proximate composition, drying was performed on all samples. The water content of all samples ranged between 29 and 30%.

#### 3.2.1. Proximate Composition

Numerous studies have been carried out on the crude protein content of yellow mealworm larvae as this has been the main reason that has attracted the interest of researchers to such a large extent.

The amount of crude protein in TM larvae varies widely based on the rearing conditions. Table 2 shows the proximate composition of TM larvae fed the different MO diets. Although the content of crude protein of larvae fed on the MO10 and MO25 diets was increased by 1.79 and 5.16%, differences were not statistically significant (*p* ≥ 0.05). However, the crude protein content of larvae fed on the MO50 diet was significantly higher compared to the control by 22.61% (*p* < 0.05). This finding may be due to the high protein content of the MO leaves (~26.3 g/100 g). In a previous study it was reported that when TM larvae were reared on bread leftovers with a protein content of 10 g/100 g, the larval protein content was lower than the control [34]. However, there is not always a standard pattern connecting the feed with the larval protein content, i.e., the higher the protein content of the feed, the higher the larval protein content. For instance, when TM larvae were fed diets supplemented with a low-protein orange sub-product (albedo), the protein content of the larvae increased with the increase in the percentage of albedo in the diet [20]. Hence, the inclusion of MO leaves in the diet of TM can be considered an effective means to increase the larval protein content. Subsequently, larvae grown on MO substrates can be considered more nutritive and beneficial for human consumption due to their increased protein content.

In addition, the amount of carbohydrates in the mealworm larvae was evaluated. Usually, this element is omitted whereas its quantity is considered relatively negligible, even though it is equally significant for human nutrition [35]. The significance of carbohydrates is high not only for human nutrition but also for insects, as through the consumption of carbohydrates they ensure sufficient energy to carry out their metamorphosis—evolution [36]. The increased protein content of the larvae fed the MO diets led to a reduced amount of carbohydrates and more specifically, there was a decrease of 3.44 (no statistically significant at *p* > 0.05), 5.23 (marginally statistically significant at *p* ≅ 0.05), and 31.77% (statistically significant at *p* < 0.05) in samples MO10, MO25, and MO50 compared to sample MO0, respectively. The carbohydrate values are shown in Table 2. Although the rate of carbohydrate reduction is high, this was not reflected in the larval growth and development during the trial. On the contrary, the larvae fed on the MO diets completed their development at the same time as larvae reared on bran, having additionally a much higher larval individual weight.

The term “crude ash” refers to the amount of mineral present in various products and as mentioned above, MO is a plant, all parts of which are rich in minerals. Based on our results, it appears that as the percentage of the MO leaves in the insect feeds increased, so did the mineral content (Table 2) of the larvae that were fed on these diets. The mineral content of the larvae fed on the MO10, MO25, and MO50 diets was statistically significantly higher (*p* < 0.05) compared to the control, being increased by 6, 9, and 202%, respectively. The crude ash value in MO0 was recorded as 0.99%, which is in accordance with the values reported by previously published studies that ranged from 0.9 to 2.2% [37,38,39]. To this end, it is concluded that MO-fed larvae could also be considered a valuable source of minerals with the notable result that the addition of MO in large quantities resulted in the development of larvae so rich in minerals that this value was recorded for the first time without causing a reduction in crude protein or significant fatty acids.

Fat and fatty acids constitute the second most abundant nutrient in the composition of TM [40]. Although fat is essential in the human diet, it should be consumed in limited quantities because numerous studies have linked cardiovascular mortality to the food consumption [41]. In the present study, the percentages of fat in larvae fed on the MO0, MO10, and MO25 diets were equal and reached 26%, while larvae fed on the MO50 diet had a fat content of 17%, significantly lower by 34.62% compared to control (Table 3). This reduced fat content in the latter sample is related to the high protein levels recorded for these larvae. Numerous previous studies have reported on the fat content of TM larvae that ranges between 24% [42] to about 34% [43,44].

As far as fatty acids are concerned, an important result was obtained concerning their quantity. Comparing the present research with previous ones, it is observed that more fatty acids were detected [20,43,45]. Palmitic acid (C16:0), oleic acid (C18:1), and linoleic acid (C18:2 ω-6) were found in significant quantities. Except for oleic acid, the concentration of which decreased in larvae fed on the MO50 diet, the concentration of the palmitic and linoleic acids was significantly (*p* < 0.05) increased compared to the control and ranged from 18.83 to 19.61% and from 28.6 to 32.87%, respectively. Although the concentration of linoleic acid was found to decrease as the percentage of MO increased in the diet, still its concentration was nearly double compared to common food products, such as chicken and egg yolk [46]. The PUFA: SFA ratio is the most commonly used index to assess the nutritional value of dietary foods, the higher its value the greater the nutritional value of the food [23]. Based on our results, the PUFA: SFA ratio decreased with the increase in the percentage of MO leaves inclusion and ranged from 2.15 (MO0) to 1.82 (MO50). This decrease may seem significant, but compared to pig, lamb, and cattle meat which have PUFA: SFA ratios between 0.13 and 0.48, or fish with a PUFA: SFA ratio ranging from 0.8 to 1.6, the PUFA: SFA ratios of the TM larvae fed the MO diets are still high, which is particularly important for cardiac health [23]. High PUFA: SFA ratio values have been also reported for another novel food, i.e., seaweeds, which range for most species from 0.42 to 2.12 [23]. For a particular species, *Gracilaria changii*, even higher values have been reported (6.96) [47]. It can therefore be concluded that compared to the other widespread novel foods, the value of the PUFA: SFA ratio in TM larvae fed the MO feeds is extremely high. Moreover, the consumption of foods with a low index of atherogenicity (IA) reduces the levels of total cholesterol and LDL-C in human blood plasma [48]. In milk, the lowest value recorded was 4.08 [49] while for the larvae of this study, much lower values were recorded which ranged from 0.35 to 0.39. Shrimps are described as marine insects. Akintola et al. [50] investigated the nutritional quality of southern pink shrimp (*Penaeus notialis*) using IA as an indicator and reported values from 0.71 to 0.82, much higher than those recorded for the larvae of the present study. Consumption of food or products with a lower index of thrombogenicity (IT) is also beneficial to human health. The values obtained in insects did not show statistical differences and ranged from 0.54 to 0.58. A study conducted on an additional novel food, algae, showed values from 0.04 to 2.94, except for *Gracilaria salicornia*, which had an IT value of 5.75 [51]. Ratusz et al. [52] analyzed the fatty acid content of 29 camelina (*Camelina sativa*) oils using the index of hypocholesterolemic/hypercholesterolemic (HH) as an indicator of nutritional quality and recorded highly elevated values from 11.7 to 14.7, when TM larvae showed values from 3.41 to 3.69. The health-promoting index (HPI) is the inverse of IA, i.e., the higher the value the better for human health, as it has a positive effect against cardiovascular diseases [23]. The amounts of HPI in the larvae were sufficiently high classifying TM larvae as a highly beneficial novel food for humans. Finally, the calculated oxidizability (COX) value was also evaluated for all samples. This value showed a very positive result as it decreased between MO0 and MO50 samples. Considering that in sunflower oil the corresponding COX value is close to six [22], 20% lower than the TM samples of our study, it is proved that they possess better oxidative stability, and thus, have a longer shelf life.

#### 3.2.2. Content of TM Larvae in Vitamins C and A

As mentioned above, MO is a very nutritious multi-purpose plant, and therefore, its leaves were used as part of the insect diet to increase the amount of vitamin C. MO0 contained 209.03 µg/g of vitamin C while the corresponding content in MO50 was 294.18 µg/g, a statistically significant (*p* < 0.05) increase of 40.73% (Table 4). Compared to a previous study where orange peel waste-albedo was used as a nutritional additive in the diet of mealworm larvae, an increase in the Vitamin C content of up to 46% was recorded, similarly to our case [20]. As such, it seems that the addition of a vitamin C rich source in the feed of the larvae can significantly increase their content in the vitamin. This can render the TM an even more desirable and nutritious food source for human consumption. In addition, consuming vitamin C-enriched TM larvae can potentially bestow some of the vitamin C beneficial properties to consumers.

Carotene and vitamin A are both regarded as beneficial for humans, as intake of the former is associated with a lower risk of various types of cancer, such as breast, lung, pancreatic, stomach, esophageal, prostate and head, and neck cancers, while the latter is a potentially supportive factor in cancer treatment [53,54]. *TM* is characterized as a low source of β-carotene and vitamin A [20], but as mentioned in the introduction the MO plant is a sufficiently rich source of both nutrients. As indicated in the results, the inclusion of MO leaves in the insect diets significantly increased the content of beta-carotene and vitamin A by 129 to 491.63% with the former value referring to MO10 and the latter to MO50. It becomes evident that with the formulation of a proper diet, TM larvae can also become a good source of vitamin A and β-carotene. The amount of vitamin A in TM larvae is comparable with the amount contained in a widely consumed protein source, i.e., chicken breast. According to Orkusz [55], chicken breast contains 6.00 µg/100 g, whereas larvae with a small addition of MO (10%) in their feed reach 7.18 µg/100 g of vitamin A and with a large addition (50%) 18.56 µg/100 g of vitamin A, a statistically significant (*p* < 0.05) increase of 19.67 and 209.33%, respectively. Finally, an additional edible insect—outside the European Union—is *A. domesticus* [56]. Adults of this cricket species contain vitamin A at values of 6.53 µg/100 g [57], a value much lower than the ones recorded for the MO10, MO25, and MO50 samples of the present study. In conclusion, the addition of even a small percentage of dried MO leaves to the diet of TM larvae transforms them into a rich source of vitamin A in comparison with both conventional human food and other edible insects. The findings contribute to the growing body of research on edible insects as a sustainable and nutritious alternative food source for human consumption.

#### 3.2.3. Antioxidant Properties of the Larvae’s Extracts

Since they provide the human organism with multiple benefits such as: lowering oxidative stress, minimizing the occurrence of health-threatening diseases, assisting with brain function and mental wellness, antioxidants are one more hot topic in the food sector. Although vitamin C and carotenoids, mainly beta-carotene, which are among the most well-known food antioxidants, were found to increase to a remarkable degree, the antioxidant activity of the larvae was also studied for more clear and thorough results. Among various properties, the MO plant shows anti-inflammatory, antioxidant, and anti-cancer activity [58,59]. In accordance with previous results, as the inclusion rate of MO leaves in the insect diet increased, characteristics such as proteins, minerals, and vitamins were also enhanced, thus assuming that a similar increase would occur in the antioxidant characteristics. The assessment of the antioxidant activity was performed through the FRAP values of the larvae (Table 5). Regarding the control sample (MO0), the ground bran seems to be easier to digest, as among the other nutrients the antioxidant activity also increased. Considering that in the same sample of larvae that consumed unground bran, the FRAP value was 119.39 ± 1.97 μmoL AAE/g [20], while in this case it was found to be 127.93 ± 3.74 μmoL AAE/g, showing a statistically significant (*p* < 0.05) increase of 7%. One additional assumption verified by our results is the effect of the increase in the polyphenol content with the increase in the percentage of MO in the diets. A previous study has reported that many dietary plant-derived polyphenolic components are more effective antioxidants in vitro than vitamins E or C [60]. The amount of total polyphenols (TPC) in the control sample was found similar to two previous studies by Antonopoulou et al. and Keil et al. [61,62].

## 4. Conclusions

To conclude, our results suggest that the inclusion of dried MO leaves in TM diets is considered highly suitable and beneficial for the feeding of TM larvae. Apart from the fact that larval growth and development were not negatively affected, the use of MO-fortified diets resulted in larvae enriched with proteins, vitamins, and minerals. It is worth pointing out that the best results in terms of larval growth and nutritional value were recorded when the dietary inclusion of MO leaves was 50%. As far as it concerns the fat and carbohydrate content of the larvae fed the MO diets, these were reduced due to their increased protein content. Nevertheless, the amount of fatty acids was sufficient and higher than the fatty acids amounts usually found in TM larvae. Moreover, the value of COX gives a significant advantage for the insect oil, as it was found to be quite stable towards oxidation, compared to commonly used oils. Finally, MO could be an advantageous nutritional addition, as it creates highly nutritious insects that could replace many conventional food sources in everyday human diets.

The strength of this study lies within its findings that suggest that the addition of MO leaves significantly increased the crude protein and vitamin content of *T. molitor* larvae. This finding suggests that the use of *M. oleifera* leaves as a feed additive has the potential to enhance the nutritional value of TM larvae and improve their suitability as a food source. Moreover, MO is known for its high productivity and resilience, requiring minimal water and land resources compared to conventional feed sources. By utilizing MO leaves as a feed additive, the study supports the concept of sustainable agriculture and promotes the utilization of underutilized plant resources. The weakness of the study is the lack of study focusing on the acceptance of TM larvae fed with MO leaves by consumers. It is crucial to consider the impact of altered feed composition on larval taste, texture, and overall acceptability. Conducting sensory evaluations and consumer acceptance studies would provide valuable insights into the potential challenges related to the organoleptic properties of the larvae.

As regards associated risks, MO leaves have the potential to cause allergic reactions in individuals who are sensitive to the plant. This risk should be carefully assessed to ensure the safety of consumers, particularly those with known allergies to MO. Finally, as regards the economical and practical feasibility of this rearing diet, establishing a sustainable and reliable supply chain for MO leaves would be crucial for cost-effective implementation. However, there are no reasons to hinder the implementation of this diet in bigger facilities. Further research, including comprehensive risk assessments and economic analyses, would contribute to a more detailed understanding of the viability and practicality of incorporating MO leaves as a feed additive for TM production.

## Figures and Tables

**Table 1:** Survival (%) of *Tenebrio molitor* larvae (± SD) fed for six weeks with wheat bran (control) (MO0) and bran fortified with different rates of *Moringa oleifera* leaves (10 (MO10), 25 (MO25), and 50% (MO50)) (*n* = 8).

Diets	1st Week	2nd Week	3rd Week	4th Week	5th Week	6th Week
ΜO0	98 ± 2	95 ± 3	95 ± 4	94 ± 4	94 ± 4 ^a^	94 ± 4 ^a^
ΜO10	99 ± 1	92 ± 3	91 ± 5	88 ± 3	87 ± 3 ^a,b^	87 ± 2 ^a,b^
ΜO25	97 ± 3	90 ± 3	87 ± 2	87 ± 3	85 ± 3 ^b^	85 ± 3 ^b^
ΜO50	98 ± 2	91 ± 2	86 ± 3	86 ± 4	86 ± 3 ^a,b^	84 ± 4 ^b^

In all cases, values represent means ± SD (*n* = 8; df = 3; *p* = 0.05). Within each evaluation interval (Week 1, 2, 3, 4, 5, and 6), means followed by the same uppercase letter are not significantly different (*p* > 0.05). Where no letters exist, no significant differences were noted.

**Table 2:** Proximate composition of *Tenebrio molitor* larvae fed for six weeks on wheat bran (control) (MO0) and bran fortified with different rates of *Moringa oleifera* leaves (10 (MO10), 17.5 (MO17.5), and 25% (MO25)) (*n* = 9).

% Composition of Dry Weight	Diets			
	ΜO0	ΜO10	ΜO25	ΜO50
Crude protein	36.79 ± 1.69 ^b^	37.45 ± 0.79 ^b^	38.69 ± 2.17 ^b^	45.11 ± 2.98 ^a^
Crude fat	26 ± 1.5 ^a^	26 ± 1.89 ^a^	26 ± 1.67 ^a^	17 ± 1.25 ^b^
Carbohydrates	33.88 ± 0.54 ^a^	32.75 ± 0.83 ^a^	32.17 ± 0.32 ^a^	25.71 ± 2.15 ^b^
Crude ash	0.99 ± 0.02 ^c^	1.05 ± 0.01 ^b,c^	1.08 ± 0.04 ^b^	2.99 ± 0.05 ^a^
Energy (kcal/100 g)	516.68 ± 29.97 ^a^	514.8 ± 23.17 ^a^	517.44 ± 22.25 ^a^	436.28 ± 27.92 ^b^

Within each line, statistically significant differences (*p* < 0.05) are denoted with different superscript letters (e.g., a–c).

**Table 3:** Fatty acid composition of *Tenebrio molitor* larvae fed for six weeks with wheat bran (control) (MO0) and bran fortified with different rates of *Moringa oleifera* leaves (10 (MO10), 25 (MO25), and 50% (MO50)) (*n* = 9).

Fatty Acid (%)	Diets			
	MO0	MO10	MO25	MO50
C8:0	0.01 ± 0	n.d. *	n.d.	n.d.
C10:0	0.05 ± 0 ^b^	0.05 ± 0 ^b^	0.05 ± 0 ^b^	0.08 ± 0.01 ^a^
C12:0	0.19 ± 0.01 ^a^	0.16 ± 0.01 ^b^	0.17 ± 0 ^b^	0.17 ± 0.01 ^b^
C14:0	2.12 ± 0.06 ^b^	2.26 ± 0.16 ^a,b^	2.32 ± 0.16 ^a,b^	2.44 ± 0.09 ^a^
C16:0	18.83 ± 1.26 ^a^	19.09 ± 0.78 ^a^	19.31 ± 1.12 ^a^	19.61 ± 0.8 ^a^
C16:1	0.48 ± 0.03 ^a^	0.42 ± 0.02 ^b^	0.34 ± 0.01 ^c^	0.5 ± 0.01 ^a^
C18:0	0.21 ± 0.02 ^c^	0.24 ± 0.01 ^b^	0.25 ± 0 ^b^	0.33 ± 0.01 ^a^
C18:1	28.6 ± 2 ^b^	30.27 ± 0.97 ^a,b^	31.06 ± 0.19 ^a,b^	32.87 ± 2.4 ^a^
C18:2 (ω-6)	48.1 ± 1.35 ^a^	46.25 ± 3.28 ^a,b^	45.42 ± 3.32 ^a,b^	42.79 ± 1.28 ^b^
C20:0	0.59 ± 0.04 ^a^	0.46 ± 0.03 ^b^	0.39 ± 0.01 ^c^	0.51 ± 0.04 ^b^
C18:3 (ω-3)	0.21 ± 0 ^a^	0.15 ± 0.01 ^b^	0.12 ± 0 ^c^	0.1 ± 0 ^d^
C22:0	0.43 ± 0.03 ^a^	0.44 ± 0.01 ^a^	0.42 ± 0.01 ^a^	0.44 ± 0.01 ^a^
C22:1	0.12 ± 0.01 ^b^	0.15 ± 0.01 ^a^	0.09 ± 0 ^c^	0.12 ± 0.01 ^b^
C24:0	0.07 ± 0 ^a^	0.07 ± 0 ^a^	0.07 ± 0 ^a^	0.04 ± 0 ^b^
*∑* SFA ^1^	22.49 ± 1.43 ^a^	22.76 ± 1 ^a^	22.97 ± 1.3 ^a^	23.62 ± 0.98 ^a^
*∑* MUFA ^2^	29.2 ± 2.04 ^b^	30.84 ± 1 ^a,b^	31.49 ± 0.2 ^a,b^	33.49 ± 2.42 ^a^
*∑* PUFA ^3^	48.31 ± 1.35 ^a^	46.4 ± 3.29 ^a,b^	45.54 ± 3.32 ^a,b^	42.88 ± 1.29 ^b^
*∑* UFA ^4^	77.51 ± 3.39 ^a^	77.24 ± 4.29 ^a^	77.03 ± 3.52 ^a^	76.37 ± 3.7 ^a^
PUFA: SFA ratio	2.15 ± 0.08 ^a^	2.04 ± 0.06 ^b^	1.98 ± 0.03 ^b^	1.82 ± 0.02 ^c^
MUFA: PUFA ratio	0.6 ± 0.03 ^c^	0.67 ± 0.03 ^b,c^	0.69 ± 0.05 ^b^	0.78 ± 0.03 ^a^
COX ^5^	5.29 ± 0.16 ^a^	5.1 ± 0.35 ^a,b^	5.01 ± 0.34 ^a,b^	4.76 ± 0.16 ^b^
IA ^6^	0.35 ± 0 ^c^	0.37 ± 0 ^b^	0.37 ± 0.01 ^b^	0.39 ± 0 ^a^
IT ^7^	0.54 ± 0.01 ^c^	0.55 ± 0.01 ^b^	0.56 ± 0.01 ^b^	0.58 ± 0 ^a^
HH ^8^	3.64 ± 0.07 ^a^	3.56 ± 0.04 ^a,b^	3.52 ± 0.05 ^b^	3.41 ± 0.03 ^c^
HPI ^9^	2.82 ± 0.03 ^a^	2.73 ± 0.02 ^b^	2.68 ± 0.04 ^b^	2.59 ± 0.02 ^c^

Within each line, statistically significant differences (*p* < 0.05) are denoted with different superscript letters (e.g., a–d). * n.d.: not detected. ^1^ SFAs, saturated fatty acids (%): SUM of C8:0, caprylic acid; C10:0, capric acid; C12:0, lauric acid; C14:0, myristic acid; C16:0, palmitic acid; C18:0, stearic acid; C20:0, arachidic acid; C22:0, behenic acid; C24:0, lignoceric acid. ^2^ MUFAs, monounsaturated fatty acids (%): SUM of C16:1, palmitoleic acid; C18:1, oleic acid; C22:1, erucic acid. ^3^ PUFAs, polyunsaturated fatty acids (%): SUM of C18:2, ω-6, linoleic acid; C18:3, ω-3, linolenic acid. ^4^ UFAs, SUM of MUFAs and PUFAs. ^5^ COX, calculated oxidizability value. ^6^ IA, index of atherogenicity. ^7^ IT, index of thrombogenicity. ^8^ HH index of hypocholesterolemic/hypercholesterolemic. ^9^ HPI, health-promoting index.

**Table 4:** Content of *Tenebrio molitor* larvae fed for six weeks with wheat bran (control) (MO0) and bran fortified with different rates of *Moringa oleifera* leaves 10% (MO10), 25% (MO25), and 50% (MO50) in vitamin C, β-carotene and vitamin A.

Diets	Vitamin C (µg/g)	β-Carotene (µg/g)	Vitamin A (µg RAE/100 g)
MO0	209.03 ± 8.12 ^c^	1.05 ± 0.09 ^c^	3.14 ± 0.27 ^c^
MO10	238.32 ± 2.53 ^b^	2.41 ± 0.14 ^b,c^	7.18 ± 0.41 ^b,c^
MO25	248.5 ± 6.44 ^b^	3.14 ± 0.29 ^b^	9.36 ± 0.88 ^b^
MO50	294.18 ± 3.62 ^a^	6.22 ± 1.47 ^a^	18.56 ± 4.38 ^a^

Within each column, statistically significant differences (*p* < 0.05) are denoted with different superscript letters (e.g., a–c). Where no letters exist, no significant differences were noted.

**Table 5:** Antioxidant properties (FRAP assay) and total polyphenols (TPC) of *Tenebrio molitor* larvae fed for six weeks with wheat bran (control) (MO0) and bran fortified with different rates of *Moringa oleifera* leaves 10% (MO10), 25% (MO25), and 50% (MO50).

Diets	FRAP (μmoL AAΕ/g)	TPC (mg GAE/g)
MO0	127.93 ± 3.74 ^d^	50.16 ± 0.86 ^c^
MO10	149.39 ± 3.28 ^c^	61.18 ± 1.43 ^b^
MO25	186.66 ± 9.53 ^b^	67.02 ± 0.88 ^b^
MO50	214.48 ± 3.5 ^a^	80.7 ± 4.1 ^a^

Within each column, statistically significant differences (*p* < 0.05) are denoted with different superscript letters (e.g., a–d).

## Data Availability

All the data are contained within the article.
